# Keratinocyte stem cells are more resistant to UVA radiation than their direct progeny

**DOI:** 10.1371/journal.pone.0203863

**Published:** 2018-09-12

**Authors:** Elodie Metral, Nicolas Bechetoille, Frédéric Demarne, Odile Damour, Walid Rachidi

**Affiliations:** 1 University Grenoble Alpes, Grenoble, France; 2 Commissariat à l'énergie atomique et aux énergies alternatives (CEA)/Institut Nanosciences et cryogénie (INAC)/SYstèmes Moléculaires et nanoMatériaux pour l'Energie et la Santé (SyMMES) /Lésions des acides nucléiques (LAN), Grenoble; 3 BTC/LSC HCL de Lyon, Lyon (France); 4 Gattefossé, 36 chemin de Genas, Saint-Priest (France); NYU Langone Medical Center, UNITED STATES

## Abstract

The epidermis undergoes constant renewal during its lifetime. This is possible due to a special population of keratinocyte stem cells (KSCs) located at the basal layer. These cells are surrounded by their direct progeny, keratinocyte progenitors or transient amplifying cells (TAs), which arise from cell division. Skin is exposed every day to sun radiation; in particular, UVA radiation penetrates through the epidermis and induces damage to KSCs and TAs. Although keratinocytes in the basal layer are the most likely skin carcinomas and/or photoaging cells of origin, surprisingly few studies have addressed the specific responses of these cells to UV radiation. In this study, we showed for the first time that keratinocyte stem cells were more resistant to UVA irradiation than their direct progeny, transient amplifying cells. Using both the MTT assay and clonogenic assay, we found that KSCs were more photo-resistant compared to TAs after exposure to different doses of UVA (from 0 to 50 J/cm^2^). Moreover, KSCs had a greater ability to reconstruct human epidermis (RHE) after UVA exposure compared with TAs. Finally, investigations of DNA repair using the comet assay showed that DNA single-strand breaks and thymine dimers were repaired quicker and more efficiently in KSCs compared with TAs. In a previous work, we showed that the same stem cell population was more resistant to ionizing radiation, another carcinogenic agent. Collectively, our results combined with other observations demonstrate that keratinocyte stem cells, which are responsible for epidermal renewal throughout life, are equipped with an efficient arsenal against several genotoxic agents. Our future work will try to identify the factors or signaling pathways that are responsible for this differential photo-sensitivity and DNA repair capacity between KSCs and TAs.

## Introduction

The epidermis is a pluristratified and differentiated epithelium composed of 90% keratinocytes. Only the basal cells of the epidermis can proliferate, which allows for its constant renewal. Along their progression to the surface, basal cells acquire different morphological and biochemical modifications, eventually leading to the *stratum corneum*. In this stratum, corneocytes, which are cells that have lost their nucleus and are filled with crosslinked material, assure the barrier function of the skin. A specific keratinocyte population, keratinocyte stem cells (KSCs), leads to the constant renewal of the epidermis throughout life. A stem cell is defined as a cell that, under normal circumstances, maintains its own population, is undiminished in function and size, and furnishes daughter cells to provide new functional cells in that tissue[1]. In the epidermis, stem cells are mainly located in hair follicles within a distinct structure known as the bulge. Hair follicle stem cells (HFSCs) are a particular stem cell population that is capable of differentiating into all epidermal lineages and thus can form all epidermal tissues and annexes. Another keratinocyte stem cell population is localized in the basal layer of the interfollicular epidermis, where they are surrounded by their direct progeny, transitory amplifying cells (TAs), that arise from their asymmetric divisions. These populations are morphologically and phenotypically similar, and the only difference between KSCs and TAs is their cell cycle activity. KSCs are quiescent and rarely divide, following a historically proposed possible asymmetrical division model [2,3]. Indeed, one stem cell gives rise to another stem cell and to a daughter cell or TA, which maintains the keratinocyte stem cell pool throughout life. Consequently, the same number of epidermal stem cells is found in the skin of both young and old individuals [4]. By contrast, TAs actively proliferate to generate an important number of keratinocytes, which quickly enter into the differentiation and migration processes after mitosis [5–7] as soon as they leave the basal layer. To date, no unique specific markers for KSCs have been identified, making them difficult to isolate. Some authors have proposed a simple way to isolate KSCs (rapid adhesion method (RA)) based on their ability to adhere faster than TA cells [8]. Others have demonstrated that a population of cells sorted by flow cytometry and following the phenotype integrin alpha 6^high^/ transferrin receptor CD71^low^ displayed a quiescent state once extracted; however, they displayed a high clonogenic and proliferative potential once cultured [9]. Moreover, these cells had a strong ability to reconstruct a pluristratified epidermis *in vivo* with a very low cellular seeding density [10] and were described as KSCs [11]. In comparison, alpha 6^high^/CD71^high^ presented characteristics similar to those shared by TAs [12,13].

The skin is continuously exposed to many external biological or environmental factors such as sun radiation, including UV radiation. Among the types of radiation, UVA and UVB penetrate through the epidermis and induce DNA damage to basal cells. Due to their strong absorption by DNA, UVB rays are known to generate photoproducts (cyclobutane pyrimidine dimers (CPDs), 6–4 photo-products (6-4PP) and Dewar isomers), leading to DNA mutations and cancers [14,15]. UVA rays are weakly absorbed by DNA and have been considered to be responsible for photo-ageing for years. Indeed, they generate strong oxidative stress (formation of reactive oxygen species, ROS), causing cellular damage to several macromolecules such as lipids and proteins in the dermis and epidermis [16–18]. Today, UVA rays also appear to be a source of DNA damage in keratinocytes, which makes this type of radiation responsible for skin cancer formation [19] and has led UVA to be recognized as a class I carcinogen [20]. Indeed, by inducing ROS production, UVA rays oxidize guanine bases, forming 8-oxo-7,8-dihydroguanine (8-oxoG) by the singlet oxygen, specifically [16,21,22]. Moreover, the hydroxyl radical, •OH, oxidizes purines and pyrimidines bases [23], but to a lesser extent [24]. The hydroxyl radical directly reacts with DNA that is situated close to its production location and induces a single-strand break (SSB) by attacking 2-deoxyribose fragments [25]. UVA radiation also leads to the formation of thymine dimers [26] by two reactions as follows: direct absorption by DNA [27] and a triplet-triplet energy transfer reaction [28]. The quantity of CPDs formed after UVA radiation is actually higher than 8-oxoG in culture cells [26,29] as well as in skin [30]. It is notable that CPD formation in UVA-exposed skin is dependent on the skin phototype [31]. Finally, UVA rays do not lead to the formation of double-strand breaks (DSB) but modulate the UVB-induced photoproduct distribution by facilitating 6-4PP isomerization into Dewar isomers [26].

Both UVA and UVB induce keratinocytes response but in a different way, leading to different skin response. Indeed, besides difference on penetration, sunburn and tanning induction as well as on carcinogenesis, only UVB radiation induces an increase in MC1R and CYP11B genes expression and in the production of CRH, ACTH and cortisol showing its implication in the local regulation of neuroendocrine activities [32,33]. This neuroendocrine system appears important to coordinate local and systemic responses to environment (via the implication of endogenous factors such as melatonin, serotonin and others) as well as by its ability to reset the body homeostasis adaptation mechanisms [34].

In this context, although KSCs are responsible for epidermal renewal throughout life, it is essential to maintain their integrity. Indeed, even if they are protected by their deep location within the basal layer in their niche [35] as well as their quiescent state to avoid replication mistakes, genomic instability can lead to their activation and thus to depletion, premature ageing and/or skin carcinomas (reviewed in [36]). Because UVA and UVB radiation can reach KSCs and induce DNA damage, it seems important to specifically characterize the KSC response to UV radiation. It has previously been reported that stem cells were more resistant to various stressors [37–40]. In the epidermis, the same trend was observed. Actually, an alpha6^+^/CD44^+^ cell population sharing similar characteristics to KSCs displayed resistance to apoptosis following UVB radiation or other genotoxic stressors [41]. Moreover, 24 h post-UVB radiation, the proportion of the alpha6^+^/p63^+^ cell population, considered to be stem cells, increased at the expense of the other populations, which decreased in proportion [42]. Interestingly, rapid adherent cells (10 min on collagen I) extracted from irradiated skin are photosensitive [43] but not in comparison to non-adherent cells. Finally, KSCs were demonstrated more resistant to ionizing radiation stress compared with TAs [44,45].

The aim of the study is first to characterize the KSC and TA responses to UVA radiation by assessing their cytotoxicity, clonogenicity and ability to reconstruct human epidermis *in vitro*. We previously showed that despite optimization of the rapid adhesion method, the rapid adherent cell population was not similar to the KSC population obtained by flow cytometry that was sorting following alpha6^high^/CD71^low^ [46]. For this reason, in this study, the responses to the UVA irradiation of human KSCs and TAs are immediately investigated from native skin after cell sorting by flow cytometry using aplah6 and CD71 labeling as well as during primary culture to investigate the cell phenotypes similar to those found *in vivo* in the basal layer of the epidermis. In the case of different sensitivities between the two populations, the second objective is to investigate whether this difference could be due to a difference in DNA damage induction or repair. The main findings show that KSCs are more photoresistant compared to their direct progeny, TA cells, partially due to their better DNA repair ability.

## Material and methods

### Keratinocyte extraction and culture

Primary cultures of keratinocytes were established from human skin obtained from patients undergoing surgery with informed consent, in accordance to ethical with ethical guidelines and declared to the French research ministry (Declaration no. DC-2008-162 delivered to the Cell and Tissue Bank of Hospices Civils de Lyon). The donors were healthy female between 20 and 35 years old. All donors were Caucasian and Phototype III or IV. The epidermis was separated from the dermis using 10 mg/mL dispase (Thermo Fisher Scientific, Waltham, MA USA). The cells were then dissociated using trypsin EDTA 0.05% (Thermo Fisher Scientific) for 12 min at 37°C and counted in Malassez after staining with trypan blue 0.04% (hospital pharmaceutical preparation).

### The isolation of Keratinocyte Stem Cells (KSCs) and Transitory Amplifying cells (TAs)

According to Kaur’s laboratory protocol, keratinocyte populations were defined on the basis of two cell-surface markers, namely, α6-integrin (α6) and CD71 [11]. Briefly, freshly extracted keratinocytes were incubated with a biotinylated mouse anti-human CD71 antibody (BD biosciences, New Jersey, USA) for 30 min at 4°C. After washing, cells were stained with R-Phycoerythrin (R-PE)-conjugated rat anti-human CD49f antibody (mAb anti-alpha6) and streptavidin allophycocyanin (SAV-APC) for 30 min at 4°C. α6^high^CD71^low^ (KSC) and α6^high^CD71^high^ (TA) populations were isolated using a MoFlo cell sorter (DakoCytomation, Glostrup, Denmark). For all experiments, an appropriate isotype-matched control, mAbs, was used to determine the level of background staining. After sorting, cells were counted, and viability was controlled using trypan blue before seeding for CFU and RHE. Validation of cell sorting and characterization of isolated cells (KSCs and TAs) were performed [45].

### UVA radiation

KSC and TA populations were seeded at 10 000 cells/cm^2^ in KSFM-medium supplemented with 1.5 ng/mL EGF, 25 μg/mL bovine pituitary extract (BPE) and 75 μg/ml of primocin. The next day, the culture medium was removed, the cells were rinsed twice with PBS, and irradiation with UVA (UVA 700L Waldmann, Germany) was performed at different doses (from 10 to 50 J/cm^2^) in PBS with the lid removed. The plates were placed to ice to prevent heating due to the UVA lamp. After radiation, PBS was replaced by fresh medium. Control sham irradiation (CSI) was used as the non-irradiated control.

### MTT assay

After cell sorting, KSCs and TAs were seeded at 10 000 cells/cm^2^ in KSFM-medium supplemented with 1.5 ng/mL EGF, 25 μg/mL bovine pituitary extract (BPE) and 75 μg/ml of primocin and subsequently irradiated the next day as previously described. Five days after UVA radiation, KSCs and TAs were rinsed with PBS and placed in MTT solution (Sigma, St Quentin Fallavier, France) at 1 mg/mL for 2 hours at 37°C. The MTT was then dissolved in DMSO for 30 min under agitation. Optical density (OD) was measured in 450 nm using a spectrophotometer. The results are expressed for each UVA dose as OD measured and as viability % vs. CSI as 100%. Experiments were performed on 3 samples from three donors.

### Clonogenic assay

After cell sorting, KSCs and TAs were seeded at a clonal density of 60 cells/cm^2^ onto inactivated human fibroblasts, which served as a feeder layer pre-seeded at 4000 cells/cm^2^ in keratinocyte medium DMEM and Ham’s F12 at a ratio of 3:1 (Thermo Fisher Scientific). This medium was supplemented with 10% fetal calf serum (Hyclone, Logan, Utah, USA), 10 ng/mL epidermal growth factor (EGF) (Sigma, St Quentin Fallavier, France), 24.3 μg/mL adenine (Sigma), 0.4 μg/mL hydrocortisone (Upjohn, Serb Laboratories, Paris, France), 0.12 IU/mL insulin (Lilly France, St Cloud, France), 2.10-9M triiodo-L-thyronine (Sigma), 10^−9^ M cholera toxin (Sigma) and antibiotics. The next day, the cells were irradiated as described previously and cultured for 14 days. The medium was changed three times a week. For clone staining, the cells were fixed and stained using rhodamine (Sigma) at 0.01 g/mL in 4% paraformaldehyde for 30 min. Holoclones, meroclones and paraclones were counted using microscope. Holoclone” was used to refer to large (> 5 mm) and homogeneous clones with regular and smooth contours whereas meroclones are intermediate sized, heterogeneous and display contours”. Paraclones are defined as smaller colonies and contains exclusively cells with a short replicative lifespan. Colony forming efficiency (CFE) was calculated as follows: CFU (number of meroclones + holoclones)/(cellular seeding density x 100). Experiments were performed on 3 samples from three donors.

### Reconstructed human epidermis (RHE)

#### Culture

After cell sorting, KSCs and TAs were seeded at 5.10^5^/cm^2^ in culture inserts 0.4 μm PCF (Millicell, Millipore) in keratinocyte medium (DMEM and Ham’s F12 at a ratio of 3:1 (Thermo Fisher Scientific)) supplemented with 10% fetal calf serum (Hyclone, Logan, Utah, USA), 10 ng/mL epidermal growth factor (EGF) (Sigma, St Quentin Fallavier, France), 24.3 μg/mL adenine (Sigma), 0.4 μg/mL hydrocortisone (Upjohn, Serb Laboratories, Paris, France), 0.12 IU/mL insulin (Lilly France, St Cloud, France), 2.10-9M triiodo-L-thyronine (Sigma), 10^−9^ M cholera toxin (Sigma) and antibiotics. The next day, they were irradiated at 10 J/cm^2^ as previously described. Cells were then cultured in a keratinocyte medium for 7 days in immerged conditions before elevation at air/liquid interface for 7 additional days in a differentiation medium (DMEM supplemented with 8 mg/mL bovine serum albumin (Sigma, St Quentin Fallavier, France), 0.12 IU/mL insulin, 0.4 μg/mL hydrocortisone, and antibiotics). Experiments were performed on samples from three donors.

#### Histology and immunohistological analysis

After 14 days of culture, reconstructed epidermis was fixed in neutral buffered formalin 4% (Diapath, Martinengo, Italy) for 24 h and embedded in paraffin. Paraffin-embedded formalin-fixed samples were then cut into 5-μm sections. After dewaxing and rehydration, sections were stained with hematoxylin—phloxin—saffron (HPS staining) for routine histology.

For immunohistological analysis, 5-μm tissue sections were incubated in 5% H_2_O_2_ / 3% normal goat serum (NGS; Jackson Immunoresearch, UK) to inactivate endogenous peroxidases. Non-specific binding was blocked in PBS containing 5% of NGS. Sections were then incubated with the following primary antibodies diluted in PBS / NGS 5% overnight at room temperature: Ki67 (Dako, France). The secondary antibody HRP-anti-mouse (Dako, France) was incubated for 1 hour at room temperature. Labeling was revealed using DAB (Dako, France), and slides were stained with hematoxylin.

Image processing and analysis were performed using the software MBF ImageJ for microscopy (Research Service Branch, US National Institute of Health, United States).

Epidermal thickness was obtained by measuring the distance between the basal lamina and the top of the epidermis excluding the stratum corneum. Conversion from numbers of pixels and μm was performed related to a scale included in the software. Nine different fields per experimental condition were quantified. Data are expressed in μm.

For proliferative capacity, the number of Ki67 positive-cells was counted in nine fields of each sample (n = 3).

### Alkaline comet assay

**Fig 1:**
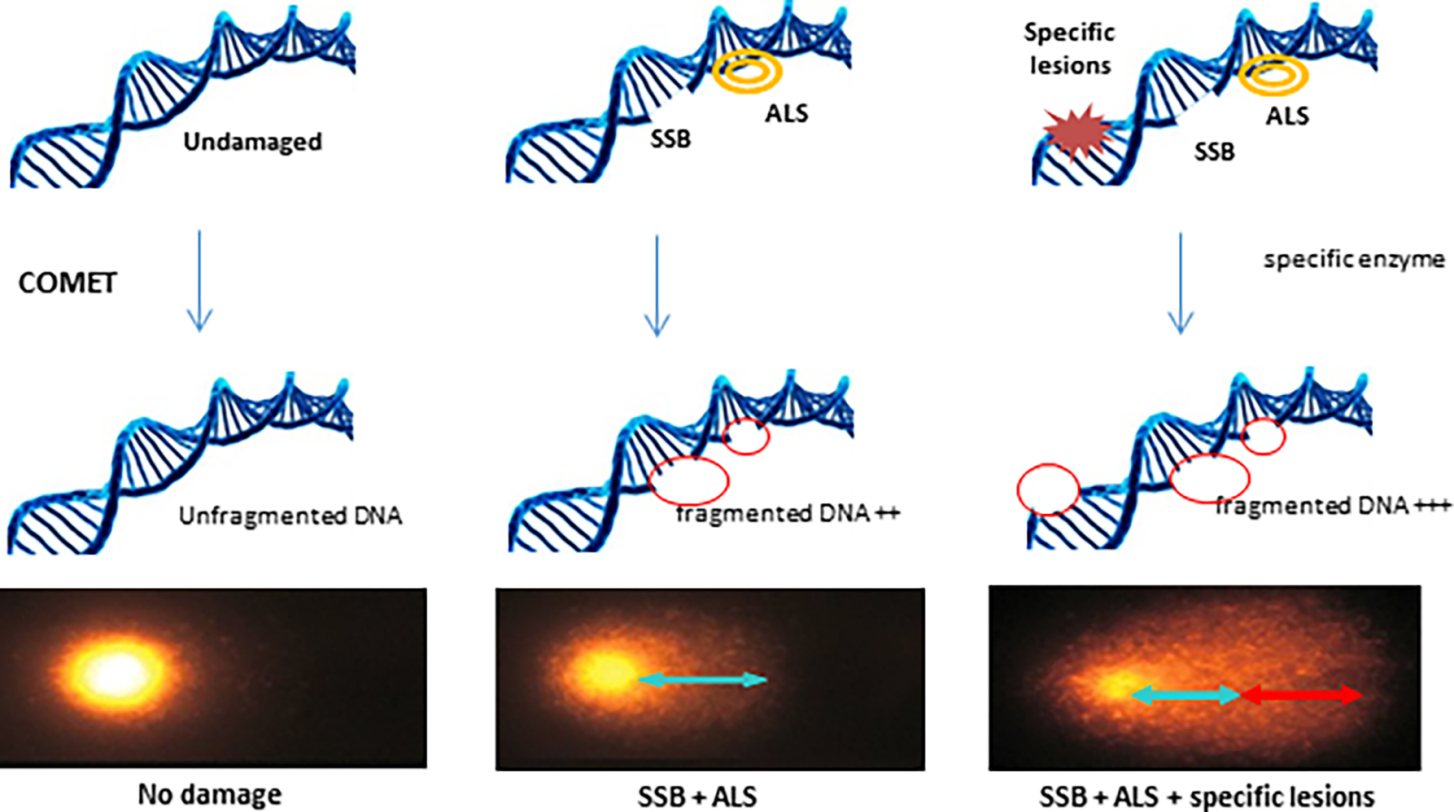
Principle of the comet assay. For revealing a specific lesion, the lesion-specific enzyme may be used to transform the lesion into a break; i.e., Fpg: enzyme cutting oxidative bases (8oxo-G et 8oxo-A) and T4 endo V: enzyme cutting CPDs.

The principle of the comet assay is shown in Fig 1. This assay allows the detection of single- and double-strand breaks as well as alkali-labile sites expressed as frank single-strand breaks in individual cells. Cell suspensions (50 μl containing 200 000 cells) were mixed rapidly with 450 μl of 0.7% prewarmed low-melting-point agarose in PBS. A total of 100 mL of the cell suspension mixture was spread on a microscope slide coated with 1% agarose and chilled to ice temperature. After gelling, the slides were treated with lysis solution [2.2 M NaCl, 89 mM ethylene-diaminetetraacetic acid disodium salt (Na-EDTA, Sigma), 8.9 mM hydroxymethylaminomethane buffer (Tris, Sigma), 30 mM N-lauroyl sarcosine, 10% dimethyl sulfoxide (DMSO, Sigma), and 1% Triton X100, pH 10] overnight at 4°C. The slides were washed three times in 0.4 M Tris pH 7.4 for 10 min and submersed in a horizontal chamber in electrophoresis buffer (0.3 M NaOH, 1 mM Na-EDTA) for 20 min at 4°C. Electrophoresis was performed at 300 mA at 4°C for 30 min. The slides were neutralized three times for 10 min with Tris buffer (0.4 M pH 7.4), stained with 50 μl of GelRed and then incubated at 4°C in a light-tight wet chamber until analysis of the slides. For revealing oxidized purines and pyrimidine dimers, Fpg (Trevigen, Gaithersburg, MD, USA) and T4endoV (BioLabs, Ipswich, MA, USA) were added after the lysis step at 0.05 U/μL for 45 min at 37°C. The slides were stained with GelRed, and comet analysis was performed using the image analysis Komet 6.0 software (Kinetic Imaging Ltd., Andor Technology plc., Belfast, Ireland). The median % tail intensity was accepted as the index of damage. For each condition, the average median % tail intensity was determined from the analysis of 150 comets (triplicate slides, 50 comets analyzed per slide) from three donors. Oxidized purines and pyrimidine dimer lesions were calculated by subtraction of damages obtained with and without Fpg/T4endoV.

### Statistical analysis

For all data, the statistical significance was assessed by the Mann-Whitney test using the software GraphPad Prism 4 (GraphPad, La Jolla, CA, USA). Statistically significant differences are indicated by asterisks as follows: * P ≤ 0.05 **, P≤ 0.005, and *** P≤0.0005. All the experiments were performed on 3 donors.

## Results

### KSC and TA responses after UVA Exposure

#### Short-term resistance against UVA-induced cytotoxicity (photo-toxicity)

**Fig 2:**
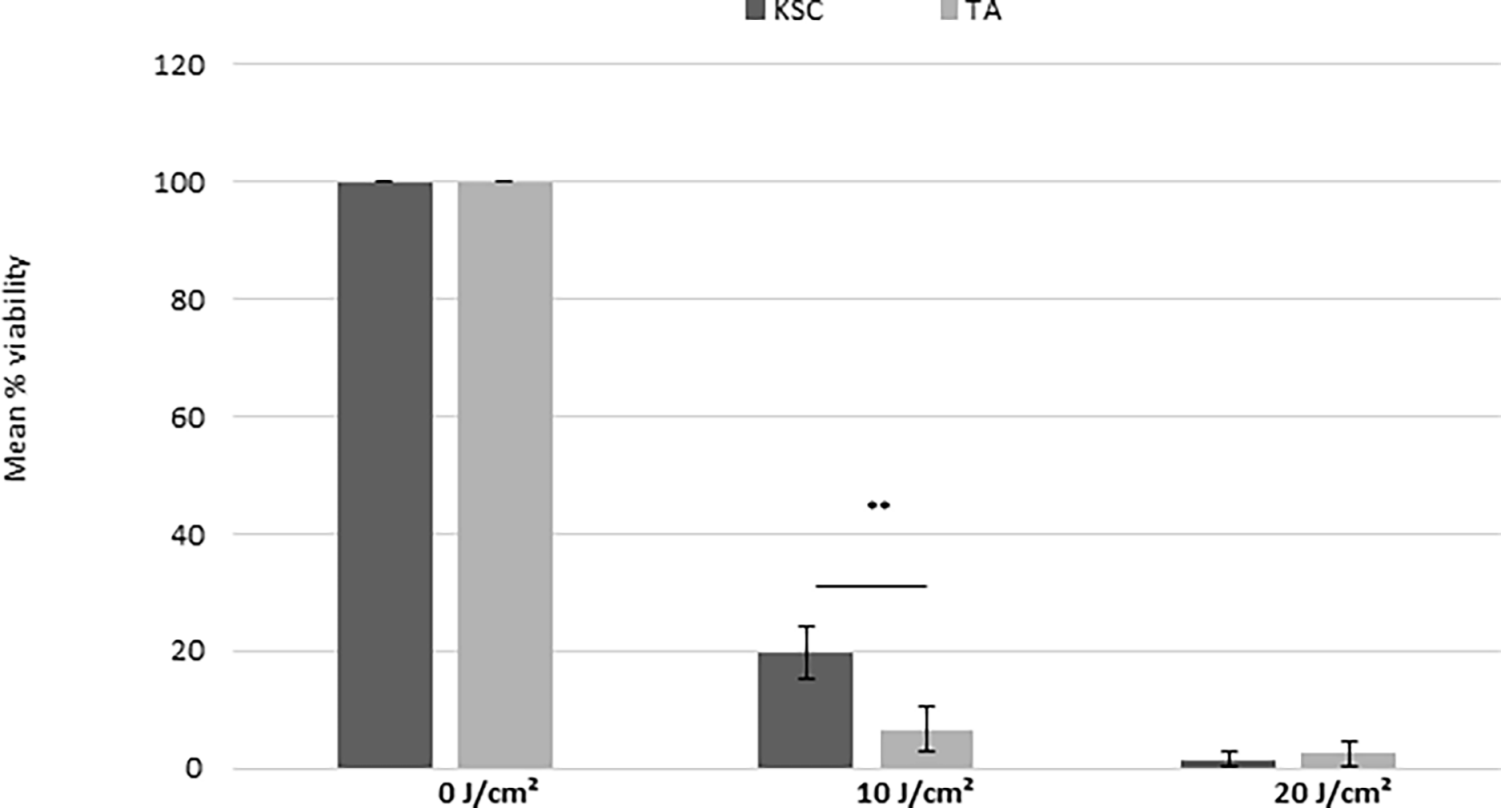
The effect of UVA radiation doses on the cytotoxicity of KSCs and TAs. The percentage of viability vs. sham irradiated cells (100%) is shown. At 10 J/cm^2^, KSCs are more resistant to UVA radiation compared to TAs; the mean of 3 donors +- SD; ** p<0.005, *** p<0.0005.

Fig 2 shows the percentage of viability of sorted KSC and TA populations irradiated with increasing doses of UVA. After exposure to 10 J/cm^2^, the viability strongly and statistically (P<0.05) decreased for both KSCs and TAs (Fig 2 A). However, at this UVA dose, the viability was significantly higher for KSCs compared to TAs (Fig 2A and 2B), demonstrating that KSCs are more resistant to UVA-induced cytotoxicity than TAs. From 20 to 50 J/cm^2^, the cell viability was equal to zero for both cell populations (data not shown).

#### Long-term photo-resistance against UVA-induced cytotoxicity

**Fig 3:**
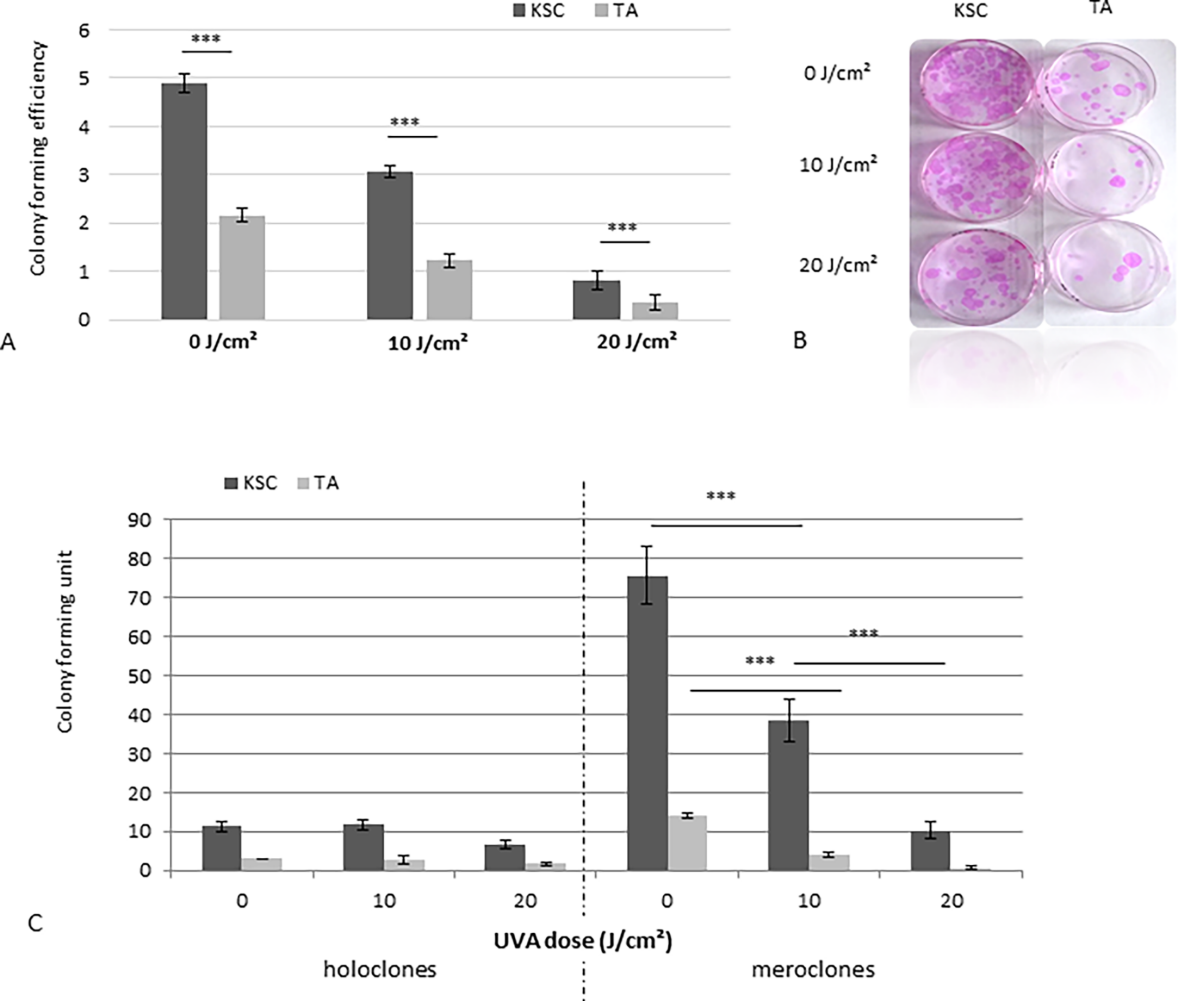
The Effect of UVA radiation doses on the clonogenicity of KSCs and TAs. A. The results are expressed as colony forming efficiency (number of colonies/seeding density x 100). At 10 J/cm^2^ and 20 J/cm^2^, KSCs form more clones compared to TAs; the mean of samples from three donors; B. A picture of clones obtained for KSCs and TAs at different UVA doses; C. Number of holoclones and meroclones for KSCs and TAs at different UVA doses; representative results of samples from 3 donors; *** p<0.0005.

The representative CFE profile of samples from three donors was obtained for KSCs and TAs irradiated at different UVA doses and is presented in Fig 3. For CSI (0 J/cm^2^), the CFE appears higher for KSCs than TAs, which shows a better clonogenic potential of KSCs, thus validating the results of the cell sorting and experiments. As described above, low doses of UVA were sufficient to induce a strong CFE reduction for both populations; however, at 10 and 20 J/cm^2^, the KSC population still displayed a higher clonogenic potential compared to TAs, which were only able to form a few colonies (Fig 3A and 3B). Interestingly, only holoclones (colonies formed by KSCs) were resistant to radiation, as shown by the strong decrease of meroclones in this irradiated TA population compared to the non-irradiated population where holoclones and meroclones were present (Fig 3C). For doses of UVA higher than20 J/cm^2^, both populations seemed to be unable to form colonies.

#### The organogenesis potential of KSC and TA populations after UVA exposure

**Fig 4:**
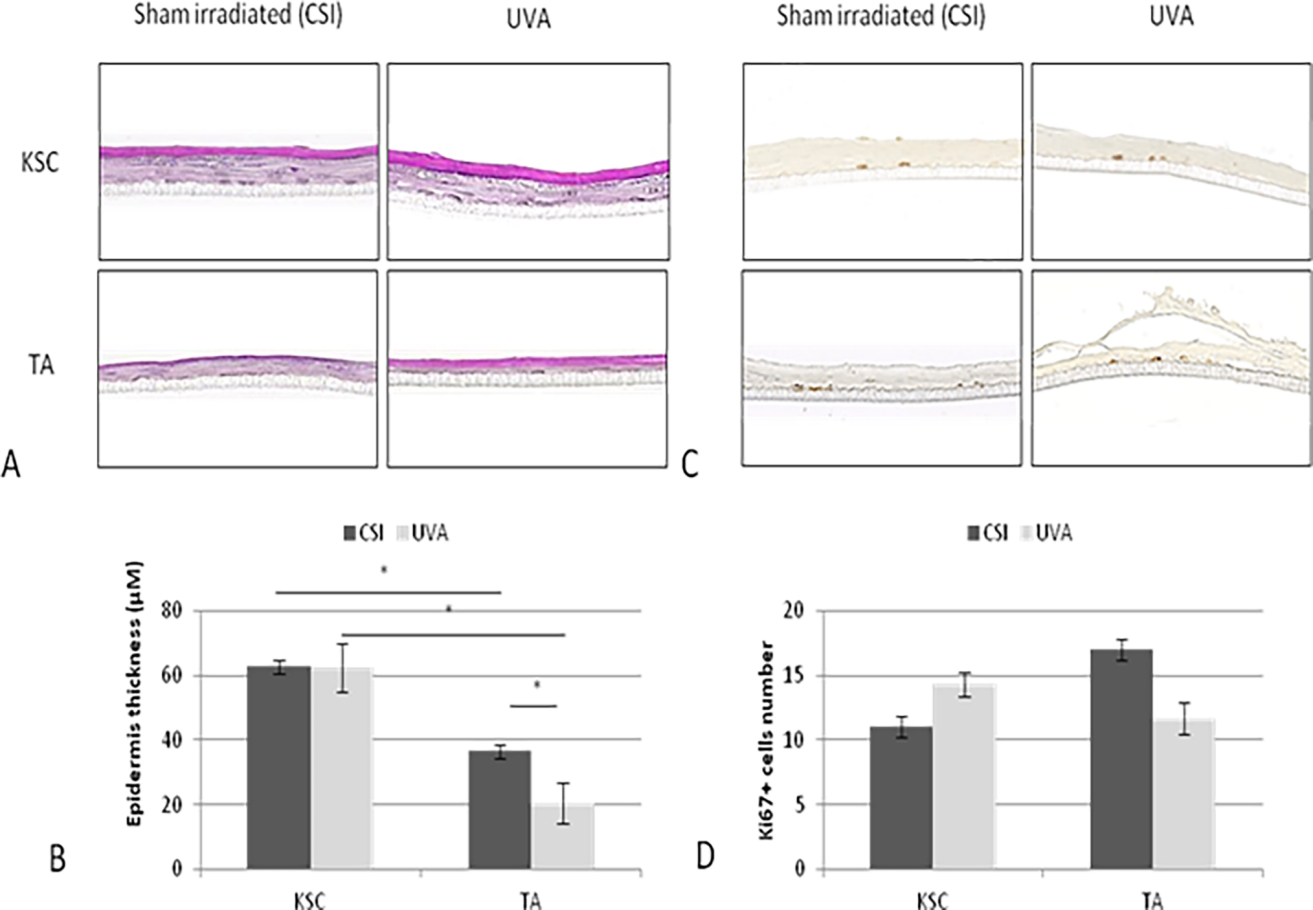
The effect of UVA radiation at 10 J/cm^2^ on the organogenesis potential of KSC and TA populations. Morphology by HPS staining (A), epidermal thickness (B), and number of Ki67+ cells (B and D) were assessed. Epidermal thickness and Ki67-positive cells were numbered on three slides per donor; representative profile of two donors;* p<0.05.

Following the results shown above, 10 J/cm^2^ was selected to test the effect of UVA radiation on the ability of KSCs and TAs to reconstruct human epidermis (RHE). Fig 4 compares RHE cultured with KSCs and TAs irradiated to 10 J/cm^2^ UVA or not irradiated (CSI). RHE cultured with non-irradiated freshly extracted keratinocytes displayed between 2 and 6 living layers of *stratum granulosum* and *stratum corneum*, which are signs of terminal differentiation (Fig 4A). The non-irradiated RHE cultured with KSCs measured 62.7 μM and is thicker than the one produced with TA cells that measured only 36.5 μM, although the number of Ki67-positive cells appeared lower in the KSC-RHE vs. the TA-RHE (11 cells compared to 17 for TA-RHE). Note that whatever the treatment conditions, a stratum corneum layer was observed for all of the reconstructed epidermis.

After UVA exposure, only KSC-cultured RHE was able to preserve its thickness and integrity. Indeed, KSC-RHE thickness appeared very similar to that of CSI, with a stabilization of approximately 62 μM, whereas the thickness of the TA-cultured RHE significantly decreased from 35 μm to 20 μm (P = 0.0286) with UVA radiation (Fig 4A and 4C). Concerning the proliferation state, after radiation, the number of proliferative cells tended to increase in the KSC-RHE, whereas they decreased in the TA-RHE.

All together, these results show that KSCs are more photo-resistant to low UVA radiation than their direct progeny, the TA cells.

### Single-strand breaks and repair of thymine dimers is faster in KSCs compared to TAs

**Fig 5:**
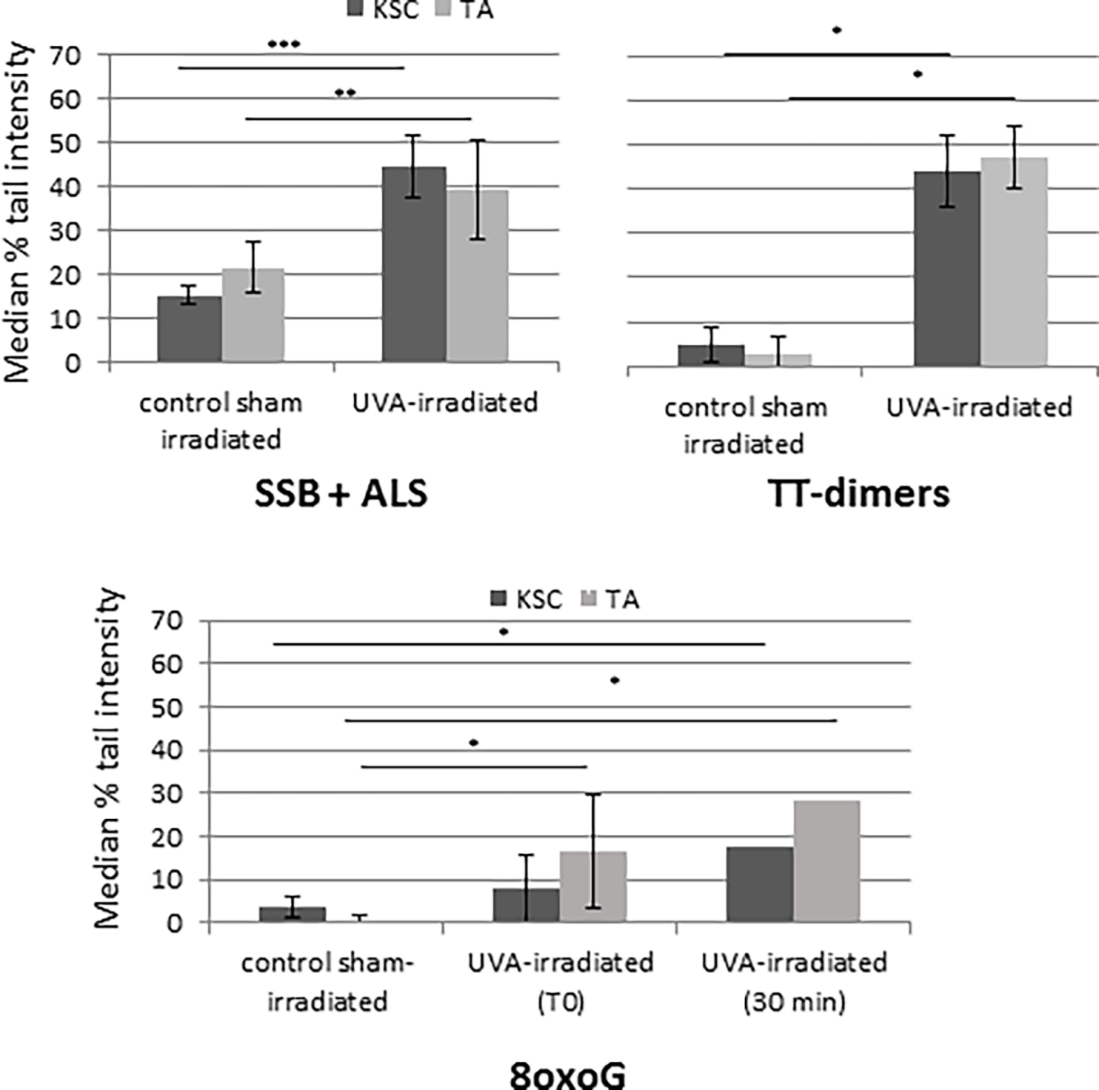
SSB, ALS, 8oxoG and thymine dimer induction in UVA-irradiated KSCS and TAs (at 10 J/cm^2^) by comet assay +- Fpg/T4 endoV. The results are expressed as the median % tail intensity. The results for 8oxoG and TT-dimers were obtained by subtraction of the comet assay results with and without Fpg and T4endoV, respectively; the mean of samples from 3 donors +- SD; * p<0.05 **, p<0.005 ***, p<0.0005.

**Fig 6:**
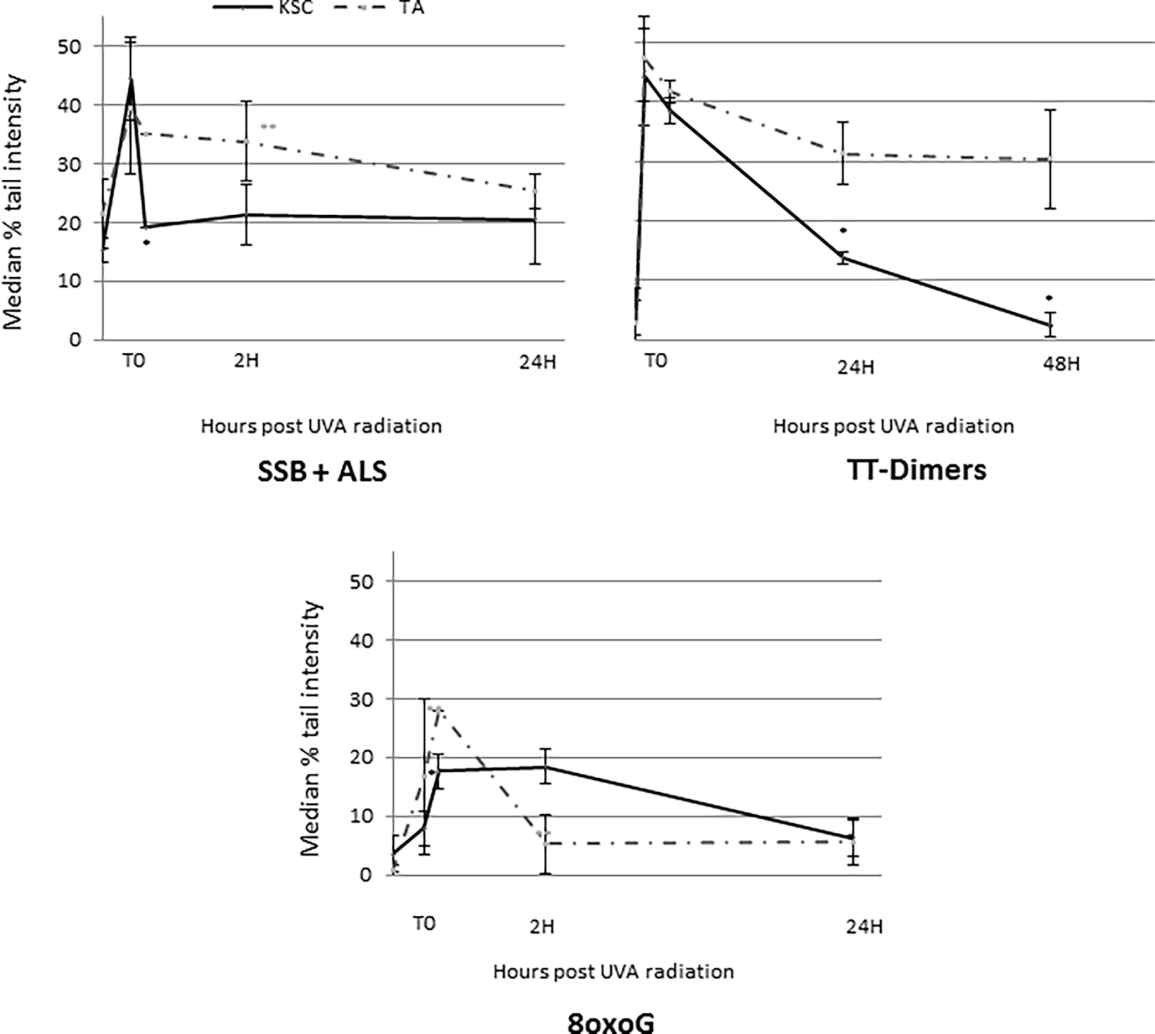
SSB, ALS and 8oxoG repair of UVA-irradiated KSCs and TAs (at 10 J/cm^2^) using comet assay +- Fpg/T4endoV. The results are expressed as the median % of remaining lesions. The results for 8oxoG and TT-dimers were obtained by subtraction of comet assay results with and without Fpg and T4endoV, respectively; the mean of samples from 3 donors +- SD. Statistical analyses were performed for each population (dotted line for TAs and black line for KSCs) between each time and the previous time; * p<0.005, ** p<0.005.

Global (SSB and ALS), oxidative (8oxoG) and pyrimidine dimer (CPDs) DNA lesion induction and repair were characterized in sorted KSCs and TAs using the alkaline comet assay with or without Fpg or T4 endoV (Figs 5 and 6). Repair kinetics was described as the disappearance of DNA lesions according to the time post-UVA exposure.

#### DNA damage induction is similar in both populations

As shown in Fig 5, non-irradiated cells (CSI) did not show any DNA lesions in either population. The peak of SSBs and CPDs is significantly higher in both irradiated populations vs. CSI and highest immediately after UVA exposure (T0) (tail intensity of approximately 30% and 40% for SSB and CPDs, respectively). For this time (T0), the levels of SSB and CPDs were similar for UVA-irradiated KSCs and TAs. Concerning 8oxoG, at T0, the level of 8oxoG appeared particularly low (tail intensity of approximately 10%) in both UVA-irradiated populations in contrast to SSB and CPD damage. For this time, there was no difference in 8oxoG damage level between the irradiated populations. However, the level of 8oxoG was significantly different for UVA-TA compared to CSI-TA, whereas it was not different for UVA-KSC compared to CSI-KSC. The peak of 8oxoG appeared 30 min after UVA-radiation and was higher for TAs than for KSCs; however, the difference was not statistically significant. For this time, the levels of 8oxoG observed in TAs and in KSCs were significantly higher than those measured in CSI-TA (p<0.005) and CSI-KSC (p<0.05).

#### DNA damage repair displays a difference between KSCs and TAs

Despite no difference in DNA damage induction between the two populations, KSCs displayed a better SSB and CPD repair capacity compared to TAs (Fig 6). Indeed, the peak of global DNA damage (SSB and ALS) was revealed by comet assay without Fpg and was highest immediately after 10 J/cm^2^ UVA radiation for both populations; however, the repair process was achieved within 30 min for the KSC-enriched population and took 24 h for TA cells to achieve. Indeed, the SSB level was significantly reduced between T0 and 30 min post-exposure in KSCs.

The peak of the CPD lesions reached the maximum immediately after radiation (T0) for both KSCs and TAs. However, for KSCs the CPD levels significantly decreased 24 h after radiation to reach the initial level at 48 h after UVA radiation. In contrast to KSCs, the level of CPDs in TAs followed a decreasing trend to 24 h post-exposure and stabilized at 48 h post UVA-exposure.

The repair of 8oxoG was faster for TAs compared to the KSC-enriched population. Indeed, 2 hours after radiation exposure, the level of 8oxoG in TAs was significantly lower than at 30 min and then returned to the initial level before radiation. For the KSC population, 2 hours after radiation exposure, the level of 8oxoG was not significantly different from the level observed at 30 min, and it took 24 h to reach the initial level. Moreover, the level of 8oxoG was very low immediately after UVA radiation (T0), which may not be sufficient to activate DNA repair systems.

## Discussion

For the first time, our results strongly demonstrate that a keratinocyte population enriched with epidermal stem cells is more resistant to UVA radiation than their direct progeny, TA cells. KSC population was more able to survive, to repair its damages and to keep its organogenesis potential than TA population after UVA irradiation. Indeed, the KSC population displayed lower short- and long-term photosensitivities compared to TA population, as demonstrated by the MTT assay; clonogenic potential; and ability to reconstruct an epidermis *in vitro*. The viability evaluated by the MTT assay appeared higher in irradiated KSCs compared to that in irradiated TAs. The clonogenic potential was also 3 times higher for KSCs than for TAs after 10 J/cm^2^ UVA radiation. Moreover, even if the clonogenicity potential was reduced for both irradiated TA and KSC populations, holoclones, known to be formed by KSCs [47], are preserved after radiation in the TA population, suggesting that epidermal stem cells (having contaminated TA cells) are more resistant. This contamination may explain the presence of clones in the TA population after 10 J/cm^2^ UVA and thus, a similar ratio was obtained between non-irradiated and irradiated cells in both populations. In addition, the thickness of the KSC-cultured epidermis, which was 3 times greater than the thickness of the epidermis produced from TAs, remained stable after UVA radiation, most likely due to a higher number of Ki67+ cells. By contrast, the number of proliferative cells had a decreasing trend in irradiated TA-RHE, which suggests the loss of their proliferative capacity after radiation, compared to KSCs that most likely preserved their ability to proliferate in the regeneration of tissue. Note that for each studied parameter, both suspensions enriched for KSCs and TAs were irradiated before entering into their cell cycle to approximate physiological conditions. These results are consistent with other studies where KSCs were more resistant to various genotoxic stressors such as UVB [41,42] and ionizing radiation [44,45].

Several mechanisms of action can explain the difference in photosensitivity between KSCs and TAs. Quiescence is the first way to resist against accumulated DNA damage and mutations that appear with repeated divisions or following exogenous stress such as UVA radiation [48]. In addition, asymmetrical division ensures the integrity of an “immortal strand” in intestinal stem cells, but not in hair follicle stem cells. This process has not been demonstrated in KSCs to date.

Second, the modulation of one or several pathways of the DNA damage response (DDR) can occur in KSCs. Among them, cells can undergo cell cycle arrest, apoptosis can be downregulated, the expression of some genes (cytokines, growth factors, intracellular pathways) can be modulated, and DNA repair systems can be more efficient. Concerning cell cycle arrest, the unchanged colony size after radiation of both KSCs and TAs obtained in our study suggest that this mechanism is not functioning. The resistance of stem cells against genotoxic stress would be the result of a specific resistance to apoptosis. Indeed, in our study, both populations did not survive high UVA doses, but in the KSC population, a higher number of cells remained alive and were still able to form colonies after low doses of UVA irradiation, which suggests a resistance to apoptosis. In accordance, the apoptosis pathway is under regulated in KSCs after ionizing radiation [44,45] and in the large bowel [49], which makes these cells prone to the development of cancers. By contrast, the elimination of damaged cells unable to repair their lesions via apoptosis represents a specific response for avoiding cell transformation in the small intestine [49]. The Embryonic Stem Cells (ESCs) are also sensitive to ionizing radiation as the G1-checkpoint is not present, thus implying the deregulation of several pathways such as p53, p21, ATM, Chk2, Cdc25A and Cdk2, which favors apoptosis [50]. After ionizing radiation in the epidermis i*n vivo*, damaged proliferative keratinocytes (TAs) are eliminated by the natural process of mitosis/differentiation [51].

Another mechanism can be the modulation of gene transcription and/or intracellular pathways. Indeed, the FGF2 pathway is upregulated in irradiated KSCs [44,45], and the Wnt/β-catenin pathway is implied in the radio-resistance of mammary stem cells [52]. Skin cells also synthetize various endogenous factors protecting the skin from DNA damage and oxidative stress. For instance, melatonin may protect DNA against free radical damage not only by modulating the gene expression of antioxidant enzymes or scavenging hydroxyl radicals but also via regulation of several key genes involved in DNA damage repair pathways [53]. Besides this melatoninergic system, a secosteroidogenic system implying the 1,25(OH)2D3, 20(OH)D3 and 20,23(OH)2D3 also protects the skin against the damage induced by UVB [54]. Note that all these aspects (cell cycle and cellular pathways) were not the main focus of this work and thus were not further investigated.

In our study, we focused on DNA damage and repair. As UVA induced strong oxidative stress, which is an accountable factor for stem cell senescence and ageing [55,56], global UVA cell sensitivity could be related to ROS production, leading to DNA damage as SSB, oxidized bases such as 8oxoG, as well as impairment of repair system enzymes [57–59]. Indeed the oxidation of OGG1 reduces the ability to repair 8oxoG [59]. In addition to these alterations, CPDs are also induced [26]. Our results showed no difference in SSB, 8oxoG and CPD induction between KSCs and progenitors immediately after UVA radiation. However, KSCs displayed a greater DNA repair capacity; SSB repair was faster for the KSC population, which was consistent with a previous report [44]. Indeed, only 30 min after UVA radiation, all SSBs were repaired, whereas it took 24 h for the SSBs to be repaired in the TA population. Moreover, KSCs also repaired CPDs faster as evidenced by a reduction in damage 24 h post-exposure and repair completed at 48 h after radiation, whereas CPDs persisted in the TA population, as shown for whole keratinocytes [30]. In accordance, better repair capacities have been reported in embryonic stem cells [60,61], iPS [62], adult neural stem cells [63,64], adult mammary stem cells [65] and KSCs [44,45].

Concerning 8oxoG, the peak of damage appeared 30 min after radiation, most likely due to a late production from the mitochondria [66] and is lower in KSCs compared to TAs, which suggests better protection from ROS. Consistently, previous reports showed that stem cells were much less susceptible to damage from hydrogen peroxide than differentiated cells [67] and in cases where ROS were decreased in a murine epidermal side-population with stem cell-like properties [68]. Such resistance has also been reported in embryonic stem cells [50], blood progenitors [67] and hematopoietic stem cells [69]. For embryonic stem cells, the mechanism of ROS production may be explained by a lower number of mitochondria that are less mature and associated with energy production via glycolysis instead of oxidative phosphorylation [70–74]. For blood progenitors, antioxidant enzymes (catalase, glutathione peroxidase and manganese superoxide dismutase) are upregulated [67]. However, such ROS protection systems have not yet been demonstrated in KSCs.

Repair system enzymes are also impaired by UVA-induced oxidative stress [57–59]. A better protection from ROS in KSCs may be linked to the higher endogenous repair capacities observed here for SSBs and CPDs. By contrast, in the TA population, 8oxoG damage appears to be more rapidly repaired (within 2 hours). We can also suppose that cells do not develop a repair system for damage that they do not undergo. Indeed, even if it has not been demonstrated, quiescent cells may not be prepared for potential damage once they enter into a proliferative cycle, as observed here for 8oxoG [75]. By contrast, oxidative damage in embryonic stem cells was reported to be more efficient [38]. However, note that due to the low initial level of 8oxoG observed here, especially in KSCs, it is particularly difficult to interpret the repair kinetics, and other studies should be performed. As the base excision repair (BER) is implicated in SSB repair and the nucleotide excision repair (NER) in CPD repair, the expression of genes implicated in BER and NER pathways could be upregulated in KSCs, in addition to cytokines or growth factors. However, due to the low cell number obtained after cell sorting, the choice of techniques to evaluate the expression of repair genes is limited, making it difficult to perform these evaluations.

Even if KSCs are more photo-resistant than TAs, they still clearly display photosensitivity. Indeed, the MTT assay results and the decrease in clonogenicity from 10 J/cm^2^ UVA radiation are possible results of accumulating genetic alterations that can compromise the genomic integrity of KSCs. In contrast to TAs that are actively proliferative but rapidly eliminated by the differentiation program, KSCs persist throughout life, making them a specific target for photocarcinogenesis. Indeed, over the long term, the genotoxic stress may induce KSC impairment, loss of function [76] and/or deprivation from their niche (reviewed by [77]) with premature ageing and/or carcinogenesis as consequences. In addition, the structure and composition of the stem cell niche is also modified by UV radiation [78,79], which implies the loss of communication between KSCs and their microenvironment, which is essential for their regulation and maintenance [80].

## Conclusions

To conclude, we demonstrated that a population enriched in KSCs is more resistant to UVA radiation than TAs, notably because of the high endogenous repair ability. Indeed, SSBs and CPDs were repaired faster in KSCs compared to TAs. Concerning 8oxoG, the peak of damage appeared to be lower in KSCs than TAs, which suggests better protection from ROS production. Finally, even if KSCs are have increased resistance to DNA damage, they still display photosensitivity and should be protected from genotoxic stress, making them a specific target for photoprotection.
